# Synergistic action of antimicrobial peptides and antibiotics: current understanding and future directions

**DOI:** 10.3389/fmicb.2024.1390765

**Published:** 2024-07-31

**Authors:** Sattar Taheri-Araghi

**Affiliations:** Department of Physics and Astronomy, California State University, Northridge, CA, United States

**Keywords:** antimicrobial resistance, antimicrobial peptides, combination therapy, antibiotic synergy, drug combination

## Abstract

Antibiotic resistance is a growing global problem that requires innovative therapeutic approaches and strategies for administering antibiotics. One promising approach is combination therapy, in which two or more drugs are combined to combat an infection. Along this line, the combination of antimicrobial peptides (AMPs) with conventional antibiotics has gained attention mainly due to the complementary mechanisms of action of AMPs and conventional antibiotics. In this article, we review both *in vitro* and *in vivo* studies that explore the synergy between AMPs and antibiotics. We highlight several mechanisms through which synergy is observed in *in vitro* experiments, including increasing membrane permeability, disrupting biofilms, directly potentiating antibiotic efficacy, and inhibiting resistance development. Moreover, *in vivo* studies reveal additional mechanisms such as enhanced/modulated immune responses, reduced inflammation, and improved tissue regeneration. Together, the current literature demonstrates that AMP-antibiotic combinations can substantially enhance efficacy of antibiotic therapies, including therapies against resistant bacteria, which represents a valuable enhancement to current antimicrobial strategies.

## 1 Introduction

Antimicrobial resistance represents a major global health challenge, threatening public health worldwide (World Health Organization et al., 2015; WHO, 2020, 2023). The emergence of multidrug-resistant bacteria has made many traditional antibiotics ineffective, limiting treatment options for infectious diseases (Rossolini et al., 2014; CDC, 2019). Innovative approaches that both enhance the effectiveness of existing antibiotics and prevent the development of resistance are crucial for addressing this growing crisis. Among these, synergistic therapeutic strategies that combine antibiotics have shown promise in combating drug-resistant infections (Lehár et al., 2009; Cokol et al., 2011; Tamma et al., 2012; León-Buitimea et al., 2020; Zhu et al., 2022).

In this context, the use of antimicrobial peptides (AMPs) in combination therapies has attracted significant attention. AMPs are natural defense molecules found in most multicellular organisms, including humans, that exhibit a broad spectrum of antimicrobial activity. Their unique “physics-based” mechanisms of action, which involve intricate electrostatic and hydrophobic interactions with lipid membrane structures, allow them to penetrate cell membranes and disrupt their integrity (Zasloff, 2002; Brogden, 2005; Gordon et al., 2005; Hancock et al., 2012; LaRock and Nizet, 2015). AMPs bind onto the membrane of their target cells and form pores across the membrane–a mechanism that is markedly different from conventional antibiotics that target specific bacterial enzymes, proteins, or pathways related to growth and proliferation (Matsuzaki et al., 1995, 1998; Epand and Vogel, 1999; Shai, 1999; Heller et al., 2000; Huang, 2000; Yang et al., 2001; Taheri-Araghi and Ha, 2007, 2010; Gualerzi et al., 2013). AMPs are known for their rapid bactericidal activity, low likelihood of resistance development, immunomodulatory properties, and potential for enhancing wound healing (Ganz, 2003; Jenssen et al., 2006; Hancock et al., 2012; Mookherjee et al., 2020).

The distinct pore-forming mechanism of AMPs made them promising candidates for combination therapies with conventional antibiotics. The outcome of combination therapy depends on various factors, including the choice of antibiotics, the target pathogen, and the environmental conditions (Greco, 1995; Chou, 2006; Cokol et al., 2011; Imamovic and Sommer, 2013; Yu et al., 2016; Grassi et al., 2017). Over recent decades, numerous *in vivo, in vitro*, and preclinical studies have explored combinations of AMPs with conventional antibiotics against various pathogens. While most published studies report synergistic effects that enhance antimicrobial activity and potentially mitigate resistancecite (Paula Jorge and Pereira, 2012; Yu et al., 2016; Grassi et al., 2017; Mhlongo et al., 2023), instances where combinations fail to exhibit synergy were also reported (He et al., 2015).

The current body of literature on AMP combination therapies, although still evolving, provides valuable insights into the mechanisms driving their synergistic action. Further research is needed to evaluate their long-term efficacy and toxicity in clinical settings.

This review article examines the existing literature on the use of AMPs in combination with traditional antibiotics. It explores the mechanisms of synergy, reviews empirical evidence from *in vitro* and *in vivo* studies, and discusses strategies to optimize these combinations. We also address the challenges and future directions in the development of these combination therapies to combat the growing threat of antimicrobial resistance.

## 2 Synergistic therapeutic approaches: AMPs and conventional antibiotics

The investigation of combination therapies include both AMP-AMP and AMP-conventional antibiotic combinations (Westerhoff et al., 1995; Mittal et al., 2016; León-Buitimea et al., 2020; Zhu et al., 2022; Mhlongo et al., 2023). Researchers have employed a variety of experimental techniques to assess the efficacy of these combinations, which can be broadly categorized into *in vitro, in vivo*, and preclinical studies (Paula Jorge and Pereira, 2012; Grassi et al., 2017). Various methodologies were used in the experimental approaches, which reflect the diverse expertise of research groups in this field, ranging from physics, chemistry, biochemistry, and biology to medical disciplines.

A significant portion of the literature focuses on *in vitro* experiments (Grassi et al., 2017; Mhlongo et al., 2023), in many cases targeting antimicrobial-resistant bacteria (Graham and Coote, 2007; Iwasaki et al., 2007; Desbois and Coote, 2011; Almaaytah et al., 2019; Morroni et al., 2019; Shang et al., 2019; Wongkaewkhiaw et al., 2019). This focus highlights a collective hope that combination therapies can address drug resistance challenges (Rossolini et al., 2014; CDC, 2019; WHO, 2023). While the knowledge on the outcomes of specific drug combinations are crucial, the broader objective is to find the underlying mechanisms that govern these interactions.

The effectiveness of AMP-antibiotic combinations is influenced by several factors besides the choice of specific AMP and antibiotic, including their concentrations and dosing regimens, the target organism, the presence and type of resistance mechanisms, and the local microenvironment (Maisetta et al., 2009; León-Buitimea et al., 2020; Zhu et al., 2022). A comprehensive understanding of these factors is essential for optimizing combination therapies and advancing the development of novel therapeutic strategies (Gordon et al., 2005; Jenssen et al., 2006; Mittal et al., 2016; Nešuta et al., 2016). Various mechanisms have been proposed to explain the synergy between AMPs and antibiotics (Grassi et al., 2017; Mhlongo et al., 2023). Detailed understanding of these mechanisms is crucial for the optimization and successful application of AMP-antibiotic combinations in combating bacterial infections. This understanding will not only facilitate the development of effective therapeutic strategies but also contribute to the broader goal of overcoming the challenges posed by antibiotic resistance (Brogden, 2005; Straus and Hancock, 2006; Hancock et al., 2012).

To enhance the antimicrobial activity of AMPs, several optimization strategies have been tested, such as peptide modification, the creation of synthetic analogs, and formulation techniques aimed at improving stability and bioavailability (Khara et al., 2015; Mittal et al., 2016; Nešuta et al., 2016). These efforts proved promising in improving efficacy of AMPs and broadening their clinical applications (Zhu et al., 2022). However, there is a need for further research to understand the long-term efficacy, toxicity, and pharmacokinetics of these therapeutic combinations (WHO, 2020, 2023).

## 3 *In vitro* studies on the efficacy of synergistic AMP combinations

**Figure 1:**
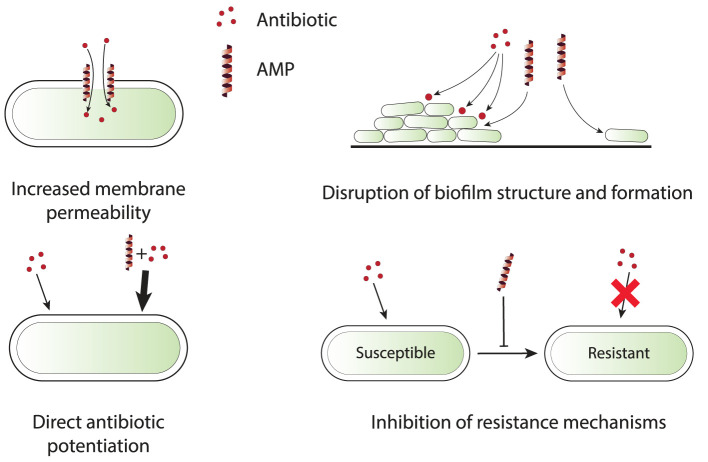
Schematic representation of four primary synergy mechanisms that are observed in *in vitro* experiments for AMP-antibiotic combinations. **Increased membrane permeability**: AMPs disrupt bacterial membrane integrity, allowing antibiotics to penetrate more efficiently, especially those targeting intracellular processes. **Disruption of biofilm structure and formation**: AMPs break down the biofilm matrix or inhibit its formation, exposing bacteria to antibiotics that can then effectively clear infections. **Direct antibiotic potentiation**: AMPs directly enhance the potency of antibiotics by modifying bacterial metabolic processes and increasing antibiotic sensitivity. **Inhibition of resistance mechanisms**: AMPs interfere with bacterial resistance mechanisms, such as efflux pumps and resistance gene expression, reducing the likelihood of resistance development and improving antibiotic efficacy.

Numerous *in vitro* studies have demonstrated the potential of combination therapy using AMPs and conventional antibiotics. *In vitro* experiments provide a controlled environment to investigate the actions of AMP-antibiotic combinations and may reveal the underlying mechanisms, including effectiveness against antibiotic-resistant strains. Table 1 presents a comprehensive list of *in vitro* studies, detailing the specific AMPs, antibiotics, and organisms tested. From these references, four main categories of mechanisms by which AMPs enhance the efficacy of antibiotics have been identified. These mechanisms include increased membrane permeability, disruption of biofilms, direct enhancement of antibiotic efficacy, and inhibition of resistance mechanisms. These categories are schematically depicted in Figure 1 and are briefly discussed in this section to provide insights into the synergistic actions that can potentially overcome antibiotic resistance in clinical settings.

**Table 1:** *In vitro* studies of AMPs combination with antibiotics.

**AMP(s)**	**Antibiotic**	**Target bacteria**	**References**
KFFKFFKFFK, IKFLKFLKFLK	Rifampin, Erythromycin, Fusidic Acid, Novobiocin	*E. coli, E. cloacae, K. pneumoniae, S. typhimurium*	Vaara and Porro, 1996
Gaegurin 6 (GGN6, PTP6, PTP12)	Chlorhexidine, Xylitol	Oral streptococci	Kim et al., 2003
Citropin 1.1	Clarithromycin, Doxycycline, Rifampicin	*R. equi*	Giacometti et al., 2005
G10KHc	Tobramycin	*P. aeruginosa*	Eckert et al., 2006
Tachyplesin III	Piperacillin-tazobactam	*P. aeruginosa*	Minardi et al., 2007
Diastereomeric AMPs	Methicillin, Cefotaxime, Tetracycline, Chloramphenicol, Rifampicin	*P. aeruginosa*	Iwasaki et al., 2007
Tachyplesin III, Colistin	Imipenem	*P. aeruginosa*	Cirioni et al., 2007
α-helical AMPs	Rifampicin	*P. aeruginosa*	Cirioni et al., 2008
Esc(1–18)	Amikacin, Colistin	*S. maltophilia*	Maisetta et al., 2009
Bacteriocin PsVP-10	Chlorhexidine, Triclosan	*S. mutans, S. sobrinus*	Lobos et al., 2009
Lactoferrin	Ciprofloxacin, Clarithromycin, Minocycline	*P. gingivalis, P. intermedia*	Wakabayashi et al., 2009
Colistin	Tobramycin	*P. aeruginosa*	Herrmann et al., 2010
Protegrin-1, PMAP-23, LL-37, Indolicidin, Cathelicidin-BF	Aureomycin	*E. coli, Salmonella*	Liu et al., 2011
Colistin, Daptomycin, Polymyxin B, Nisin	Lysostaphin	*S. aureus*	Desbois and Coote, 2011
Pleurocidin	Erythromycin, Chloramphenicol, Ampicillin	*S. aureus, E. faecium, P. acnes, E. coli, P. aeruginosa*	Choi and Lee, 2012
Coprisin	Ampicillin, Vancomycin, Chloramphenicol	*S. aureus, E. faecium, P. acnes, E. coli, P. aeruginosa*	Hwang et al., 2013
PMAP-36, PRW4	Aminoglycoside antibiotics	*E. coli, S. aureus*	Wang et al., 2014
Various α-helical AMPs	Imipenem, Cefepime, Levofloxacin Hydrochloride, Vancomycin	*S. aureus, S. pneumoniae, Staphylococcus epidermidis, P. aeruginosa, E. coli, K. pneumoniae*	Feng et al., 2015
Plectasin	β-lactams, Aminoglycosides, Glycopeptides	*S. aureus*	Hu et al., 2015
Various α-helical AMPs	Rifampicin	*M. smegmatis*	Khara et al., 2015
Azithromycin	Colistin	*P. aeruginosa, K. pneumoniae, A. baumannii*	Lin et al., 2015
HYL and analogs	Rifampicin	*S. aureus, P. aeruginosa*	Nešuta et al., 2016
CLP-19	Ampicillin, Ceftazidime, Erythromycin, Levofloxacin	Various drug-resistant bacteria	Li et al., 2017
SPR741	Azithromycin, Clarithromycin, Erythromycin, Fusidic Acid, Mupirocin, Retapamulin, Rifampin, Telithromycin	*E. coli, K. pneumoniae, A. baumannii*	Corbett et al., 2017
Synthetic peptides	Ciprofloxacin, Meropenem, Erythromycin, Gentamicin, Vancomycin	*E. faecium, S. aureus, K. pneumoniae, A. baumannii, P. aeruginosa, E. cloacae, E. coli*	Pletzer et al., 2018
PrAMPs (A3-APO, ARV-1502)	Imipenem, Colistin, Meropenem, Ceftazidime	*K. pneumoniae, A. baumannii, E. coli*	Otvos et al., 2018
Trp-containing AMPs	Penicillin, Ampicillin, Erythromycin	*S. epidermidis*	Shang et al., 2019
Protegrin 1, ChBac3.4, defensins, LL-37, lysozyme	Gentamicin, Ofloxacin, Oxacillin, Rifampicin, Polymyxin B, Silver nanoparticles	Various Gram-positive and Gram-negative strains	Zharkova et al., 2019
AamAP1-Lysine	Levofloxacin, Ampicillin, Chloramphenicol, Rifampicin, Erythromycin	*S. aureus, P. aeruginosa*	Almaaytah et al., 2019
SLAY-P1	Vancomycin	*Enterococcus*	Liu et al., 2019
Protegrin-1	Colistin, Fosfomycin, Levofloxacin, Meropenem, Tigecycline, Rifampin	*A. baumannii*	Morroni et al., 2019
Various AMPs containing the RWQWR motif	Ciprofloxacin, Vancomycin	*E. coli, P. aeruginosa, S. aureus, E. faecalis*	Vargas-Casanova et al., 2019
D-LL-31	Ceftazidime	*Burkholderia pseudomallei*	Wongkaewkhiaw et al., 2019
Melimine, Mel4, Protamine	Cefepime, Ciprofloxacin	*P. aeruginosa, S. aureus*	Kampshoff et al., 2019
WLBU2, BMAP-18, Mastoparan, Nisin, Melittin, Magainin II, Bactenicin, CAMA	Tigecycline, Minocycline, Novobiocin, Tetracycline, Fosfomycin, Ceftazidime	*B. anthracis, Y. pestis, F. tularensis, and B. mallei*	Cote et al., 2020
SLAP-S25	Cefepime, Colistin, Ofloxacin, Rifampicin, Tetracycline, Vancomycin	*E. coli*	Song et al., 2020
LL-37	Colistin	*E. coli*	Morroni et al., 2021
Pt5-1c	Oxacillin, Vancomycin, Streptomycin, Azithromycin	*S. aureus, E. coli, K. pneumoniae*	Duan et al., 2021
CEP-136	Rifampicin, Clarithromycin, Azithromycin	*E. coli, K. pneumoniae, A. baumannii, P. aeruginosa*	Mood et al., 2021
Nisin, P10	Ceftazidime, Tobramycin, Ciprofloxacin, Doripenem, Colistin	*A. baumannii, P. aeruginosa*	Jahangiri et al., 2021
Nal-tagged peptides	Vancomycin	Various resistant bacteria	Wu et al., 2021
Various AMPs from a peptide library	N/A	*S. aureus*	Maron et al., 2022
GVF27	Ciprofloxacin	*Burkholderia cepacia* complex	Bosso et al., 2022

### 3.1 Increased membrane permeability

AMPs compromise bacterial membrane integrity, thus enhancing the penetration and efficacy of antibiotics. Specifically, AMPs enable antibiotics that target intracellular processes to reach their sites of action more efficiently. This action is crucial against bacteria equipped with efflux pumps that actively expel antibiotic molecules. For instance, the synthetic peptide β-Ala-modified analogs of anoplin demonstrate significant membrane disruption, followed by enhanced antimicrobial potency and synergistic effects with conventional antibiotics against drug-resistant *Pseudomonas aeruginosa*, without prompting resistance development (Zhong et al., 2020).

Similarly, the antimicrobial peptide LL-37, when combined with colistin, showed strong synergy, drastically reducing the minimum inhibitory concentrations (MIC) against multidrug-resistant *Escherichia coli*. This highlights LL-37's role in membrane permeabilization and efflux pump circumvention (Morroni et al., 2021). In another study, pleurocidin was found to enhance antibiotic effectiveness by inducing hydroxyl radical formation, which contributes to membrane damage, as well as by disrupting bacterial cytoplasmic membranes, thereby promoting antibiotic entry (Choi and Lee, 2012).

Cathelicidin peptides also demonstrated such mechanisms by disrupting bacterial cell membranes and enhancing the bactericidal activity of co-administered antibiotics such as aureomycin against enteric pathogens (Liu et al., 2011). In similar synergistic interactions, the peptide coprisin not only exhibited intrinsic antimicrobial properties but also enhanced the activity of conventional antibiotics by facilitating their access to internal bacterial targets, crucial for combating biofilm-forming bacteria (Hwang et al., 2013).

The combined use of peptides PMAP-36 and PRW4 with aminoglycosides also showcased the role of membrane permeabilization, where these peptides enhance the intracellular delivery of antibiotics, contributing to a synergistic antibacterial effect (Wang et al., 2014).

These findings collectively highlight the critical role of AMPs in disrupting bacterial membranes, which not only enhances the efficacy of antibiotics against resistant strains but also broadens the therapeutic window of existing antimicrobial agents. This provides a robust strategy against infections that are difficult to treat with traditional antibiotics alone.

### 3.2 Disruption of biofilms

AMPs have demonstrated an ability not only for disrupting the biofilm matrix but also for inhibiting the formation of biofilms in the first place. As such, AMPs can facilitate the action of antibiotics by exposing otherwise protected bacteria within a biofilm structure, thus enhancing their susceptibility to treatments. This mechanism is particularly important in addressing persistent infections.

The peptide Cecropin A has been shown to disrupt uropathogenic *E. coli* biofilms, enhancing the efficacy of the antibiotic nalidixic acid, and leading to a synergistic clearance of infections without inducing resistance, a vital aspect in treating recurrent infections (Kalsy et al., 2020). Similarly, the peptide GVF27 targets biofilms formed by *Burkholderia cepacia* complex, known for its robust antibiotic resistance, enhancing the effects of traditional antibiotics like ciprofloxacin (Bosso et al., 2022).

In another study involving both *in vitro* and *in vivo* experiments with murine subcutaneous abscess model, the combination of synthetic peptides with meropenem and erythromycin significantly reduced infection sizes caused by ESKAPE pathogens (*E. faecium, Staphylococcus aureus, K. pneumoniae, Acinetobacter baumannii, P. aeruginosa, and Enterobacter* spp), which are notorious for their biofilm-forming capabilities and multidrug resistance (Pletzer et al., 2018).

Additionally, peptide Pt5-1c, when combined with vancomycin and streptomycin, has shown to not only disrupt biofilms but also restore antibiotic sensitivity in multidrug-resistant strains, offering a dual advantage (Duan et al., 2021). The LL-37 peptide has also been reported to significantly reduce biofilm formation and, in combination with colistin, shows enhanced bactericidal activity against multidrug-resistant bacteria (Morroni et al., 2021).

The AMP-antibiotic synergy against biofilms have also been observed citropin 1.1, which, when combined with rifampicin, shows enhanced activity against *Rhodococcus equi* biofilms (Giacometti et al., 2005). Furthermore, combinations of early generation antibiotics with AMPs have been effective against biothreat agents like *Burkholderia mallei* and *Yersinia pestis*, indicating the potential of these combinations in both clinical applications (Cote et al., 2020).

### 3.3 Direct antibiotic potentiation

AMPs can also directly augment the potency of conventional antibiotics through mechanisms that modify bacterial metabolic processes and increase antibiotic sensitivity. For instance, the peptide CLP-19 was reported to synergistically enhance the effects of both bactericidal and bacteriostatic agents. It significantly reduces the adverse effects associated with antibiotic-induced endotoxin release, which is especially important in severe infections (Li et al., 2017).

Additionally, studies have shown that some AMP SPR741 can potentiate antibiotics by circumventing bacterial resistance mechanisms, such as efflux pumps in *E. coli*, thus enabling higher intracellular concentrations of antibiotics (Corbett et al., 2017).

In another example, SLAP-S25, a peptide incorporating non-natural amino acids, has a minimal antibacterial effect on its own but substantially increases the efficacy of a broad range of antibiotics against multidrug-resistant pathogens. This enhancement is due to its ability to act alongside antibiotics in overcoming bacterial defenses (Song et al., 2020).

Antibiotic azithromycin was shown to have enhance activity when used in combination with AMPs. Although traditionally not recommended for multidrug-resistant Gram-negative infections, azithromycin showed significant bactericidal activity when combined with colistin, pointing to potential against difficult-to-treat infections (Lin et al., 2015).

The combination of AMPs and conventional antibiotics not only holds promise *in vitro* but has also proven effective *in vivo*, significantly reducing infection levels and suggesting a viable strategy for enhancing the efficacy of existing antibiotics. This approach may help mitigate the development of antibiotic resistance and improve treatment outcomes for infections caused by resistant bacteria (Kampshoff et al., 2019).

### 3.4 Inhibition of resistance mechanisms

Some studies have reported the role of AMPs in inhibiting antibiotic resistance through various mechanisms, which is crucial for managing drug-resistant infections (Maron et al., 2022). This offers mechanisms that traditional antibiotics cannot exploit. For instance, proline-rich AMP A3-APO, in combination with colistin, was shown to significantly improve treatment efficacy and hindered resistance in treated pathogens (Otvos et al., 2018). This synergy is also seen in other combinations, where AMPs and antibiotics together achieve a greater antimicrobial effect and reduce the likelihood of resistance development (Zharkova et al., 2019).

Further, the AMP Cecropin A disrupts uropathogenic *E. coli* biofilms and inhibits efflux pump activity, the two mechanisms that induce bacterial resistance. Cecropin A was shown to slow the emergence of resistance while clearing infections (Kalsy et al., 2020). Similarly, β-Ala modified peptides like Ano-1β and Ano-8β exhibit strong membrane disruption and are noted for their low propensity for resistance development compared to standard antibiotics (Zhong et al., 2020).

Moreover, novel AMPs like CSM5-K5 not only exhibit potent bactericidal activity but also restore antibiotic sensitivity in previously resistant strains. Such findings are crucial, as they show that AMPs can reverse resistance trends-a significant advantage in the current era of high antibiotic failure rates (Thappeta et al., 2020).

## 4 *In vivo* studies on the efficacy of synergistic AMP combinations

**Figure 2:**
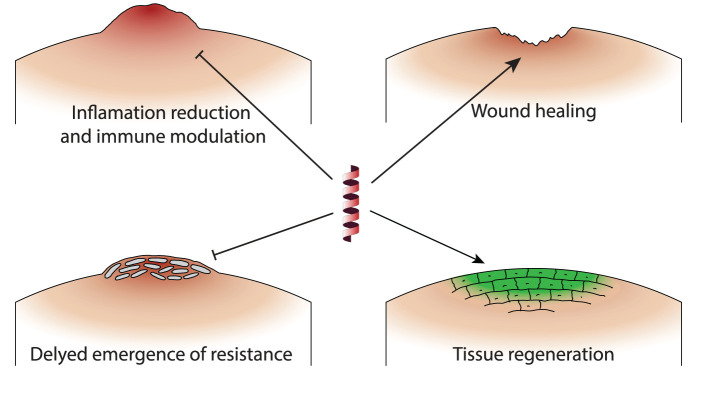
Schematic representation of the primary mechanisms by which AMP-antibiotic combinations demonstrate unique effects in *in vivo* studies. **Inflammation reduction and immune modulation**: AMPs enhance immune responses and reduce inflammation, promoting monocyte/macrophage migration to infection sites. **Enhancing wound healing**: Certain combinations prevent biofilm formation and support tissue regeneration, beneficial for infections with indwelling medical devices. **Delayed emergence of resistance**: AMPs sustain antibiotic efficacy and delay the development of resistant bacterial strains, extending the usefulness of antibiotics. **Tissue regeneration**: AMP-antibiotic combinations reduce MRSA burden in wound and systemic infections, demonstrating enhanced tissue regenerative properties.

The translation of *in vitro* synergistic effects of AMP-antibiotic combinations to *in vivo* models represents a crucial step in the development of effective therapeutic strategies against infectious diseases. In the complex biological systems of living organisms experimented with in *in vivo* experiments, certain synergistic mechanisms not identifiable in *in vitro* studies come to light, providing additional insight into how these combinations might be optimized for clinical use. Table 2 presents a list of *in vivo* studies with information on the AMP-antibiotic combinations that were tested, as well as target bacteria and the animal model. Figure 2 highlight four mechanisms identified in *in vivo* experiments which are related to the host organism, thus not available to study in *in vitro* settings. In this section, we briefly discuss these mechanisms and their implications for enhancing the effectiveness of AMP-antibiotic therapies in clinical applications.

**Table 2:** *In vivo* studies of AMPs combination with antibiotics.

**AMP(s)**	**Antibiotic(s)**	**Target bacteria**	**Animal Model**	**References**
Tachyplesin III	Piperacillin-tazobactam	*P. aeruginosa*	Rat ureteral stent infection model	Minardi et al., 2007
α-helical peptides	Rifampicin	*P. aeruginosa*	Rat models	Cirioni et al., 2008
Colistin	Rifampicin	*P. aeruginosa*	Mouse pneumonia model	Aoki et al., 2009
Ranalexin	Lysostaphin	*S. aureus*	Rabbit wound infection, mouse systemic infection	Desbois et al., 2010
Colistin	Tobramycin	*P. aeruginosa*	Rat lungs	Herrmann et al., 2010
Cathelicidin peptides	Aureomycin	*E. coli, Salmonella*	Weaning piglets	Liu et al., 2011
PL-5	Levofloxacin hydrochloride	*S. aureus*	Mouse infection model	Feng et al., 2015
Plectasin	β-lactams, Aminoglycosides, Glycopeptides	*S. aureus*	Murine models	Hu et al., 2015
Trp-containing AMPs	Penicillin, Ampicillin, erythromycin	*S. epidermidis*	Mouse infection model	Shang et al., 2019
SLAY-P1	Vancomycin	*Enterococcus*	Galleria mellonella	Liu et al., 2019
Synthetic peptides	Ciprofloxacin, meropenem, erythromycin, gentamicin, vancomycin	Mixed ESKAPE pathogens	Murine sub-cutaneous abscess model	Pletzer et al., 2018
CEP-136	Rifampicin, azithromycin	*E. coli, K. pneumoniae, A. baumannii, P. aeruginosa*	Murine peritonitis model	Mood et al., 2021
Random Peptide Mixtures	Random peptide mixtures	*A. baumannii*	Mouse models of acute pneumonia and soft tissue infection	Caraway et al., 2022
PMAP-36	Tetracycline, Gentamicin	*E. coli*	Murine model	Tao et al., 2023

### 4.1 Inflammation reduction and immune modulation

In *in vivo* settings, AMPs have been observed to enhance immune responses, a mechanism not tested in *in vitro* experiments. For example, the combination of PMAP-36 and tetracycline not only reduced bacterial load but also promoted the migration of monocytes/macrophages to the site of infection, significantly increasing survival in murine models (Tao et al., 2023).

Another unique *in vivo* mechanism involves the reduction of inflammation and cytokine production. Certain AMPs, when combined with antibiotics, have demonstrated a significant decrease in inflammatory markers and cytokine levels, contributing to better clinical outcomes. This effect is particularly beneficial in treating infections where inflammation exacerbates the disease process, such as in cystic fibrosis or sepsis (Aoki et al., 2009; Herrmann et al., 2010).

### 4.2 Delayed emergence of resistance

An important advantage of using AMPs in combination with antibiotics is the delayed emergence of bacterial resistance. *In vivo* studies demonstrate that AMPs can sustain antibiotic efficacy and reduce the evolution of resistant strains, providing a sustainable approach to managing bacterial infections. For instance, PMAP-36 has been shown to delay the development of resistance to tetracycline in porcine extraintestinal pathogenic *E. coli*, highlighting its potential as part of a combination therapy to extend the usefulness of existing antibiotics (Hu et al., 2015; Tao et al., 2023).

### 4.3 Enhancing wound healing and tissue regeneration

Beyond their antimicrobial action, some AMP-antibiotic combinations have been shown to possess wound healing and tissue-regenerative properties. Minardi et al. (2007) demonstrated that Tachyplesin III, when combined with piperacillin-tazobactam, prevented *P. aeruginosa* biofilm formation on ureteral stents in a rat model, highlighting the potential for preventing infections in patients with indwelling medical devices. Desbois et al. (2010) explored the synergistic effect of ranalexin with lysostaphin in wound and systemic infections, showing significant reduction in MRSA burden in both rabbit and mouse models.

## 5 Optimization strategies for enhancing synergy between AMPs and conventional antibiotics

Several methods have been used to optimize the efficacy of AMPs and their synergistic action with antibiotics. Optimization is essential for enhancing the collective efficacy of drug combinations, reducing the potential for resistance development, and minimizing toxicity. Here, we outline some of the strategies utilized to enhance the efficacy of AMP-antibiotic combinations.

### 5.1 AMP modification for enhanced stability and specificity

Modifying the structure of AMPs is a fundamental approach to enhance their utility in combination therapies. Strategies such as amino acid substitution, cyclization, and the incorporation of non-natural amino acids are employed to improve stability, membrane penetration, and specificity. Such modifications are designed to optimize the interaction of AMPs with bacterial membranes, thereby enhancing their antimicrobial effectiveness while reducing potential cytotoxic effects on mammalian cells (Giacometti et al., 2005; Taheri-Araghi and Ha, 2007; Choi and Lee, 2012; Khara et al., 2015; Mittal et al., 2016; Nešuta et al., 2016).

### 5.2 Tailored combination therapy based on pathogen profile

Tailoring therapy to the specific pathogens and their resistance mechanisms can significantly enhance the efficacy of AMP-antibiotic combinations. This precision medicine approach involves selecting AMPs and antibiotics that complement each other's mechanisms of action and are effective against the resistance profiles of the target bacteria. Consideration of the local microenvironment at the infection site is crucial to this strategy. Although still in its infancy, such targeted approaches hold great promise for improving therapeutic outcomes (Yu et al., 2016; Zhu et al., 2022).

### 5.3 Utilization of high-throughput screening and computational models

To further refine optimization strategies, high-throughput screening methods and computational modeling serve as essential tools. These techniques allow for the rapid identification of effective AMP-antibiotic pairs by predicting potential synergies based on the properties of the drugs and the characteristics of the target bacteria. This can significantly accelerate the development of effective combination therapies (Maisetta et al., 2009; Paula Jorge and Pereira, 2012; Mookherjee et al., 2020).

### 5.4 Bioinformatics tools and AMP databases

The use of bioinformatics tools and comprehensive databases that catalog information on AMPs supports the discovery and design of novel AMPs. These resources are invaluable for researchers seeking to develop new synergistic combinations that can be effectively integrated into clinical practice (Maisetta et al., 2009; Paula Jorge and Pereira, 2012).

These strategies represent a multifaceted approach to enhancing the synergy between AMPs and conventional antibiotics. Each method contributes to a deeper understanding and more effective application of these combinations in the battle against resistant infections. As research progresses, these optimization strategies will likely evolve, offering new avenues for combating antimicrobial resistance and optimizing therapeutic strategies.

## 6 Future directions

Research into synergistic therapeutic approaches involving AMPs and conventional antibiotics has made significant progress in recent years, which presents a promising strategy against infections, including resistant bacteria. Both laboratory (*in vitro*) and live subject (*in vivo*) studies have demonstrated the efficacy of AMP-antibiotic combinations against a wide range of pathogens, including drug-resistant strains and infections associated with biofilms (Tables 1, 2 and references therein). These studies have also demonstrated a range of mechanistic models that lead to the synergistic action of that enhance antimicrobial effectiveness, suppress the emergence of resistance, and even promote wound healing and tissue regeneration (Sections 3 and 4).

However, despite these advances, there are several challenges and limitations to the future development of AMP-antibiotic combination therapies. Firstly, while *in vitro* assays offer valuable insights into the benefits of AMP-antibiotic synergy, translating these findings into *in vivo* models and further in clinical settings is highly complex. More extensive preclinical assessments and clinical trials are needed to confirm the safety, efficacy, and pharmacokinetics of these combination therapies in human subjects (Seyhan, 2019; Dijksteel et al., 2021; Talapko et al., 2022).

Furthermore, substantially more detailed understanding of the mechanisms driving synergistic interactions is necessary to inform the design of effective combination therapies for treating infectious diseases. Precise mechanistic knowledge would predict the expected outcomes of combination therapy. Among various methodologies, high-throughput screening and computational models can expedite the identification of optimal AMP-antibiotic pairs and forecast synergistic effects. This knowledge would enable us to refine optimization strategies for enhancing synergy and mitigating resistance development. Peptide engineering, dosage optimization, and tailored delivery mechanisms for different pathogens and resistance profiles hold great potential but require further refinement and validation (Tan et al., 2021; Zhang and Yang, 2022).

Addressing biofilm-related infections remains paramount. While AMP-antibiotic combinations have shown promise in combating biofilms, further research is needed to confirm the efficacy of AMPs against biofilms, as some studies have highlighted limitations in their effectiveness (Taheri-Araghi and Guerbidjian, 2020). Similarly, efforts to overcome drug resistance and expand the use of AMP-antibiotic combinations are ongoing. Besides studies reporting the lack of resistant development against AMPs, there is also evidence that microorganisms have developed resistance to certain AMPs (Perron et al., 2006).

In conclusion, research on AMP-antibiotic combinations offers hope for combating antimicrobial resistance. However, addressing remaining challenges and guiding future research will be essential to fully realize their potential in treating infections. Collaborative efforts and innovation are key to revolutionizing infectious disease management and reducing antimicrobial resistance worldwide.

## Author contributions

ST-A: Investigation, Writing – original draft, Writing – review & editing.
